# The *Escherichia coli* Cell Division Protein and Model Tat Substrate SufI (FtsP) Localizes to the Septal Ring and Has a Multicopper Oxidase-Like Structure

**DOI:** 10.1016/j.jmb.2008.12.043

**Published:** 2009-02-20

**Authors:** Michael Tarry, S.J. Ryan Arends, Pietro Roversi, Evan Piette, Frank Sargent, Ben C. Berks, David S. Weiss, Susan M. Lea

**Affiliations:** 1 Department of Biochemistry, University of Oxford, OX1 3QU, UK; 2 Sir William Dunn School of Pathology, University of Oxford, South Parks Road, Oxford OX1 3RE, UK; 3 Department of Microbiology, University of Iowa, Iowa City, IA 52242, USA; 4 College of Life Sciences, University of Dundee, Scotland DD1 5EH

**Keywords:** Tat, twin arginine translocation, GFP, green fluorescent protein, T1, mononuclear type I, LB0N, LB without 10g NaCl per liter, NCS, non-crystallographic symmetry, SufI, FtsP, Tat, cupredoxin, X-ray crystallography

## Abstract

The *Escherichia coli* protein SufI (FtsP) has recently been proposed to be a component of the cell division apparatus. The SufI protein is also in widespread experimental use as a model substrate in studies of the Tat (twin arginine translocation) protein transport system. We have used SufI-GFP (green fluorescent protein) fusions to show that SufI localizes to the septal ring in the dividing cell. We have also determined the structure of SufI by X-ray crystallography to a resolution of 1.9 Å. SufI is structurally related to the multicopper oxidase superfamily but lacks metal cofactors. The structure of SufI suggests it serves a scaffolding rather than an enzymatic role in the septal ring and reveals regions of the protein likely to be involved in the protein–protein interactions required to assemble SufI at the septal ring.

## Introduction

Currently, around 20 cell division genes have been identified in the bacterium *Escherichia coli* (reviewed in Refs. 1–6). Most of these genes have the designation *fts*, which stands for ‘filamentation temperature sensitive’ and reflects the fact that the first *E. coli* division genes were discovered in screens for mutants that divided normally at 30 °C but became filamentous and ultimately died at 42 °C. The *fts* genes share two critical properties. Firstly, loss-of-function mutations cause division defects. Secondly, the proteins encoded by these genes all localize to the midcell where they form a structure called the septal ring (also termed the divisome or septalsome). Many investigators regard the septal ring as an organelle that mediates cytokinesis. It assembles at the midcell prior to division and then constricts as division proceeds so as to remain at the leading edge of the developing septum.

Samaluru *et al.* have recently shown that the *E. coli* gene *sufI* has several properties of an *fts* gene.^7^ They have proposed renaming the gene *ftsP* to emphasize its role in division and to avoid confusion with *suf* genes involved in assembly of iron–sulfur clusters.^7^ *sufI* was originally identified as a multicopy suppressor of an *ftsI*(Ts) mutation, with the *sufI* designation standing for ‘suppression of *ftsI*(Ts)’.^8^ FtsI, also known as penicillin-binding protein 3, is a transpeptidase involved in synthesis of peptidoglycan cell wall during cell division.^9–12^ Reddy and colleagues demonstrated several additional division-related phenotypes associated with *sufI*, the most noteworthy being that a *sufI* null mutant is filamentous when grown at elevated temperature in media lacking osmotic protectants such as NaCl.^7,13^ Thus, *sufI* acts like an *fts* gene, but whether it is part of the septal ring that mediates cell division remains to be determined.

SufI is a water-soluble, periplasmic protein.^8^ The sequence of SufI shows that it is a member of the multicopper oxidase superfamily.^14^ However, the SufI sequence conserves only 2 of the 12 canonical copper-binding residues found in multicopper oxidases and this has led to the prediction that SufI does not bind metal cofactors.^15^

In addition to its involvement in cell division, SufI is of considerable interest due to its use as a model substrate in studies of the bacterial Tat (twin arginine translocation) protein transport pathway. The Tat system is used to transport folded substrate proteins from the cytoplasm to the periplasm across the cytoplasmic membrane (reviewed in Refs. 16–19). SufI has a number of favourable properties for mechanistic studies of Tat transport relative to that of other *E. coli* Tat substrates, including that it is water soluble, monomeric, and does not require insertion of a cofactor during folding. These properties have led to the adoption of SufI as the preferred model substrate in Tat translocation studies in *E. coli*.^15,20–30^

In this study, we examine whether SufI/FtsP resembles other Fts proteins in localizing to the division septum. We also determine the structure of SufI/FtsP with the aim of providing additional insight into the molecular function of the protein, as well as a structural basis for studying the interactions of SufI with other cell division proteins and with the Tat translocation apparatus.

## Results

### Fusion of SufI to green fluorescent protein

Recent genetic evidence^7,13^ has demonstrated a role for SufI in protecting and stabilizing the divisional assembly under conditions of stress. Specifically the evidence implies that SufI may be a septal ring protein.

**Fig. 1:**
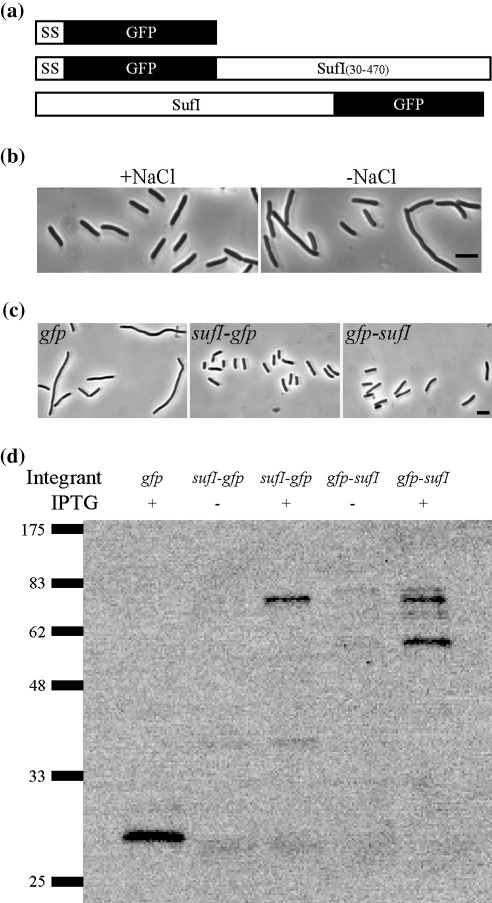
(a) Cartoon of *gfp* constructs. “SS” refers to a Tat signal sequence from TorA. SufI is a Tat substrate and therefore does not need an artificial signal sequence. (b) A *sufI* null mutant has mild division defects. EC1751 (*sufI<>aph*) was grown at 37 °C in LB with or without 1% NaCl, fixed, and photographed under phase contrast microscopy. The scale bar represents 5 μm. (c) GFP/SufI fusions rescue division defects. Derivatives of EC1751 (*sufI<>aph*) expressing the indicated *gfp* construct were grown in LB0N and photographed as above. The strains shown are EC1874, EC1875 and EC1876. The scale bar represents 5 μm. (d) Immunoblot of strains that express *gfp*, *sufI-gfp* or *gfp-sufI* under the control of an IPTG-inducible promoter. Sample concentrations were normalized by OD_600_, except that fourfold more of the *sufI-gfp* sample was loaded because this fusion gene is not highly expressed. The blot was probed with anti-GFP serum. The strains shown are *gfp* (EC1874), *sufI-gfp* (EC1876) and *gfp-sufI* (EC1875). Molecular mass markers are indicated at the left.

To test this hypothesis, we sought to develop green fluorescent protein (GFP)-tagged versions of SufI that would allow the subcellular localization of SufI under normal growth conditions to be determined. To this end we constructed separate IPTG-inducible fusions of *gfp* to the 5′ or 3′ ends of *sufI* (Fig. 1a). We term the protein products of these genes GFP-SufI and SufI-GFP, respectively. The fusion constructs were integrated into the chromosome in a single copy at the attachment site for phage φ80 in both a wild-type and a *sufI<>aph* null mutant background. Isogenic strains that expressed *gfp* alone were constructed as controls. It is important to note that GFP does not function in the periplasm unless exported via the Tat system.^31–33^ Because SufI is a Tat substrate, no precautions had to be taken to direct the SufI-GFP fusion to the Tat pathway. However, for the GFP-SufI protein and for GFP alone, a Tat signal sequence from TorA was incorporated at the N-terminus of the protein during the cloning steps.^34^

The functionality of the fusion proteins was assessed by determining whether they could complement the division defects of a *sufI<>aph* null mutant (obtained from the Keio collection^35^). When grown in standard LB containing 10 g NaCl per liter, the *sufI* mutant exhibited normal morphology, consistent with earlier reports.^7,8,13^ However, when grown in the absence of NaCl (LB0N medium), about 20% of the cells exhibited division defects that resulted in the appearance of short filaments or chains of up to three to four cells (Fig. 1b and Table 1).^7^ Division defects were more pronounced at 37 °C than at 30 °C and were confirmed after eviction of the Kan^r^ cassette to create a markerless *sufI<>frt* deletion strain (not shown). Introduction of either the *gfp-sufI* or *sufI-gfp* fusions into the *sufI<>aph* strain ameliorated the division defect observed in LB0N, with the *sufI-gfp* fusion giving essentially complete complementation (Fig. 1c and Table 1). Thus, both GFP fusion constructs retain the functionality of SufI. Immunoblotting these strains with anti-GFP revealed major cross-reactive bands at 75 kDa consistent with the calculated mass of 75 kDa for the SufI/GFP fusions after removal of their Tat signal peptides (Fig. 1d). The immunoblot of GFP-SufI also revealed a band at about 60 kDa. Analysis of purified SufI (described below) shows that a surface loop is susceptible to clipping between residues 307–308. Cleavage at this site would produce a truncated GFP-SufI(307) with a predicted mass of 58 kDa, which correlates well with the size of the GFP-containing fragment observed in the blot. It is important to note that clipping of the surface loop does not affect the integrity of the SufI protein because the two fragments remain associated, and it is therefore reasonable to assume that the clipped protein is transport competent and functional.

### SufI is localized to the septal ring

**Fig. 2:**
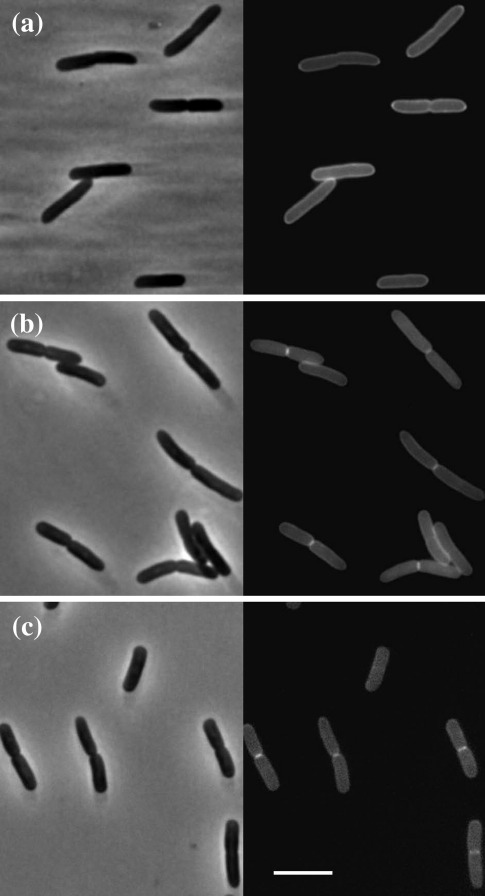
Septal localization of SufI. Phase (left) and fluorescence (right) micrographs of an *sufI<>aph* null mutant expressing (a) *gfp*, (b) *gfp-sufI* and (c) *sufI-gfp*. The scale bar represents 5 μm. The strains shown are EC1874, EC1875 and EC1876. They were grown in LB with 1% NaCl and 1 mM IPTG at 30 °C.

The fusion proteins were tested for septal localization by fluorescence microscopy of live cells on an agarose pad. SufI-GFP and GFP-SufI clearly accumulated at the division site in about half of the cells (Fig. 2 and Table 2). This was true regardless of whether the cells were grown in LB containing or lacking NaCl (Table 2). Almost all of the cells exhibiting septal localization of SufI had readily apparent constrictions. Septal localization was not observed when cells were fixed with paraformaldehyde/glutaraldehyde (not shown). In fixed cells, only uniform periplasmic fluorescence was observed. Sensitivity to fixation has been observed previously with other periplasmic division proteins such as AmiC.^34^

Division mutants sometimes have an enlarged periplasmic space. This can lead to the enrichment of periplasmic GFP signal at division sites even when the GFP fusion protein in question does not localize to the septal ring.^36^ To address this concern, we also localized periplasmic GFP that was not fused to another protein. As expected, isolated GFP was more uniformly distributed throughout the periplasm than GFP fused to SufI (Fig. 2). This finding argues that septal localization of the GFP/SufI fusions is not an artifact and that any division defect in the *sufI* null mutant is too subtle to result in an enlarged periplasmic space (at least under these growth conditions and with this assay). It should also be noted that GFP-SufI and SufI-GFP also clearly localized to the midcell in a wild-type background, which would not be expected to have an enlarged periplasmic space (Table 2).

The periplasmic location of Tat-targeted GFP in Fig. 2 is evident from the fluorescent halo around the cells. Because neither GFP-SufI nor SufI-GFP showed a similar halo, it was not immediately obvious that those proteins were also periplasmic, although the complementation and septal localization data implied that both fusion proteins had to have reached the periplasm. We suspected the lack of a fluorescent halo in cells producing GFP-SufI or SufI-GFP was due to the low abundance of these proteins as evident from Western blotting (Fig. 1d). Indeed, when GFP-SufI and SufI-GFP were produced from multicopy plasmids, the expected halos were readily apparent (Supplemental Fig. 1). Moreover, in a Tat mutant, both GFP-SufI fluorescence and SufI-GFP fluorescence were exclusively cytoplasmic and septal localization was not observed (Supplemental Fig. 2). Taken together, these observations confirm that GFP-SufI and SufI-GFP were exported to the periplasm by the Tat system. Because we were concerned that the chaining phenotype of Tat mutants^37^ might obscure septal localization, we also examined GFP fusions to the well-characterized division proteins FtsZ, FtsI and FtsN. All three localized to potential division sites (Supplemental Fig. 2). Interestingly, production of GFP-FtsI in the Tat mutant inhibited division, while production of GFP-FtsN appeared to promote more frequent division.

### Recruitment of SufI to the septal ring depends on FtsN

**Fig. 3:**
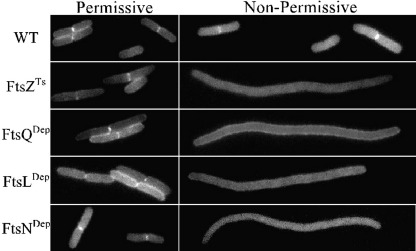
Recruitment of SufI to septal rings requires other Fts proteins. The top two panel sets show a wild-type (WT) strain or *ftsZ*(Ts) mutant grown at 30 °C in LB (permissive) or 37 °C in LB0N (non-permissive). The strains shown are EC1873 and EC2065, both of which carry a chromosomal *sufI-gfp* fusion. Traditionally, 42 °C has been used as the non-permissive temperature for *ftsZ84*(Ts), but we found that SufI-GFP was not fluorescent at 42 °C. The bottom three panel sets show FtsQ, FtsL or FtsN depletion strains. The strains shown are JM265, JOE170 and EC1908 and carry the *sufI-gfp* plasmid pDSW932. These strains produce the indicated Fts protein from an arabinose-dependent promoter and were grown in the presence of arabinose (permissive) or glucose (non-permissive). The cells shown are representative. For quantitative data, see Supplemental Table 1.

The septal ring contains 10 proteins that are essential for cell division. Studies of how these essential proteins depend on one another for recruitment to the septal ring have established a set of relationships that suggests the ring assembles by sequential addition of proteins, starting with FtsZ and ending with FtsN (reviewed in Refs. 3, 5 and 6). To determine where SufI fits into this hierarchy, we examined localization of SufI-GFP in filamentous cells that could not divide because FtsZ, FtsQ, FtsL or FtsN had been either inactivated or removed by depletion. Notably, loss of any one of these division proteins prevented SufI-GFP from accumulating at potential division sites (Fig. 3 and Supplemental Table 1). These results indicate that SufI is a late recruit to the septal ring.

### Crystal structure of SufI

In an attempt to gain further functional insights about SufI, the structure of overproduced, hexahistidine-tagged SufI was determined by X-ray crystallography. SufI crystallized in two crystal forms (Table 3) and was solved by molecular replacement using the structure of the homologous *E. coli* CueO protein [Protein Data Bank (PDB) ID 1PF3]^38^ as the search model. Both the orthorhombic and monoclinic crystal forms contain two molecules in the asymmetric unit, with a common dimer interface surface of 921 Å calculated by the MSD-PISA server.^39^ SufI elutes from a size-exclusion chromatography column at the position expected for a monomer. In addition, analysis of the dimer interface with the MSD-PISA server gave a complexation significance score of zero. This implies that the interface is not significant and most likely arises from crystal packing.

**Fig. 4:**
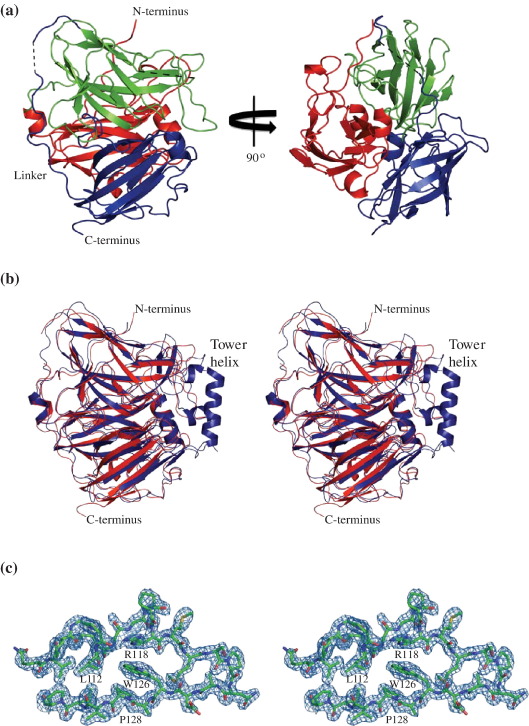
Structure of SufI. (a) Cartoon representation of SufI. The structure shown is for chain A of the orthorhombic space group with domain 1 shown in red, domain 2 in green and domain 3 in blue. Regions of missing density are shown as black dotted lines. The arrow in the region of missing density indicates where the protein is subject to proteolytic cleavage. (b) Stereo view showing CueO (blue) overlaid on the orthorhombic SufI (red) structure. PDB ID 1KV7 was overlaid onto the orthorhombic chain A of SufI with the program CCP4-Lsqkab. The positions of the N- and C-termini of the proteins are shown and the tower region of CueO is labelled for reference. The orientation is as in (a). (c) Stereo view showing representative electron density (2*F*_o_ − *F*_c_) of orthorhombic chain A, residues 107–131 contoured at 1σ. The positions of the invariant residues leucine 112, arginine 118, tryptophan 126 and proline 128 are labelled. The images were generated with Pymol.^40^

The two molecules of each asymmetric unit are essentially the same within experimental error (rmsds over 408 and 406 C^α^ atoms of 1.1 and 0.9 Å for the orthorhombic and monoclinic crystals, respectively). Of the two data sets, the orthorhombic crystals diffracted to higher resolution (1.9 Å compared to 2.6 Å). The overall structure of SufI (Fig. 4a) is very similar to that of CueO and contains three cupredoxin-like domains. These domains consist of a beta sandwich made up of seven strands in two beta sheets, although some variations may have one or two additional strands. The beta sheets are found in variations of the Greek key beta barrel, which have some of the beta strands adjacent in space despite not being adjacent in sequence. As with CueO, SufI has a long linker peptide between domains two and three that runs around the surface of the molecule.

Due to proteolysis, the SufI preparation used for crystallization is missing the first 17 amino acids of the 27-amino-acid N-terminal Tat signal peptide. The remaining 10 amino acids of the signal peptide are not ordered in any of the crystal forms. Previous circular dichroism studies of the *E. coli* SufI signal peptide have shown that it lacks a defined secondary structure in aqueous solution,^41^ which suggests that even if the full-length signal peptide were present it would be disordered. Other residues missing from the final model are listed in Table 3. In all the models, there is missing density for residues 296–311, which include the region where SufI is susceptible to proteolytic cleavage (above). No electron density was observed for the hexahistidine affinity tag, with the exception of the arginine residue of the affinity tag linker in chain A of the orthorhombic structure. Figure 4c shows electron density representative of the quality of the data and phases.

The main areas of difference between the two crystal forms lie between residues 42–47, 55–66 and 326–334. Residues 42–47 are found in a loop that leads into the first domain, while residues 326–334 compose the long linker between domains 2 and 3. The electron density for both of these regions was poor and often missing entirely for the side chains. This suggests that these differences between the two crystal forms arise from flexibility of the molecule in these regions. Residues 55–66 are found in an extended loop in the first domain: a short alpha-helical segment is present in this loop in the orthorhombic form, which is unravelled in the monoclinic form.

### SufI belongs to the multicopper oxidase family of proteins but does not bind copper

The crystal structure coordinates of a SufI monomer were submitted to the DALI server.^42^ The closest structural relative identified by the DALI server is *E. coli* CueO, with a *Z*-score of 53. An overlay of the two structures (Fig. 4b) has an rmsd of 1.46 Å over 406 C^α^ atoms.

CueO contains four copper atoms, which are found in a mononuclear type I (T1) copper centre and a trinuclear copper centre. SufI lacks all but two of the copper-binding residues found in CueO and has been assumed not to bind copper.^15^ Consistent with this prediction, no electron density corresponding to copper atoms was observed in the SufI structure. The absence of bound copper or other metals in the SufI structure was confirmed by calculating an anomalous difference density Fourier map using CCP4-FFT, which failed to identify any significant peaks.

The ability of SufI to bind metals could not be ruled out due to the use of metal chelate affinity chromatography during the purification protocol, which might remove bound metal ions from the protein. However, soaking of SufI crystals with a cocktail of metal ions did not result in the identification of any metal-binding sites, and using the CHED server^43^ to detect/predict metal binding sites also failed to identify any putative sites.

**Fig. 5:**
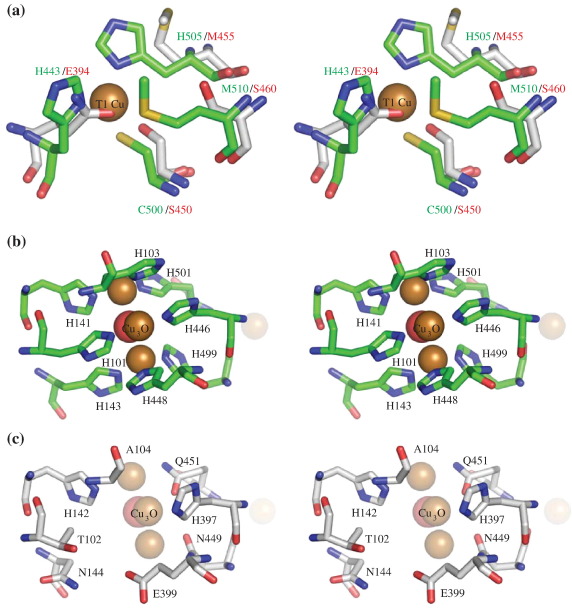
Comparison of the copper-binding sites of CueO and the equivalent regions of SufI. (a) Stereo view of the copper-binding residues of the T1 copper-binding site of CueO (green sticks) overlaid on the corresponding region of SufI (white sticks). The structure of CueO (PDB ID 1KV7) was overlaid on the orthorhombic chain A of SufI with the program CCP4-Lsqkab. The residues are labelled in green for CueO and red for SufI. The T1 copper of CueO is shown as a space-filling sphere. (b) Stereo view representation of the trinuclear copper-binding site of CueO showing the copper-binding residues as stick models. The trinuclear centre is shown as space-filling spheres. (c) The corresponding residues of SufI shown as sticks with the corresponding position of the trinuclear centre of CueO shown as transparent space-filling spheres. The structure of CueO (PDB ID 1KV7) was overlaid on the orthorhombic chain A of SufI with the program CCP4-Lsqkab. The images were generated with Pymol.^40^

In CueO, the T1 centre is slightly buried within domain 3.^38^ Figure 5a shows the T1 copper centre in CueO overlaid onto the corresponding region in SufI. This illustrates how the N and S donor ligands favoured by copper have been almost totally replaced by O donor ligands in SufI. The trinuclear copper centre of CueO is located between domains 1 and 3 (Ref. 44) and a water-filled cavity is found at the equivalent position in SufI. In CueO, the trinuclear centre is coordinated by eight histidine residues (Fig. 5b). All but two of these histidine residues have been replaced in SufI with amino acids that would not normally be expected to coordinate copper (Fig. 5c). The two histidine residues retained in SufI are residues that in CueO ligate two different atoms of the trinuclear copper centre. In the SufI crystal structures these two histidines are bridged by a water molecule.

A further difference between the CueO and SufI structures is the absence, in SufI, of the methionine-rich helix and loop, the so-called tower of the CueO structure. This region sits over the T1 copper site in CueO^38,45^ and constitutes an additional copper-binding site.^44^ Taken together, these limited sequence differences are sufficient to destroy the ability to bind copper, leading to the unusual situation of highly homologous proteins with very different biological functions.

### Identification of potential functionally important regions of SufI

**Fig. 6:**
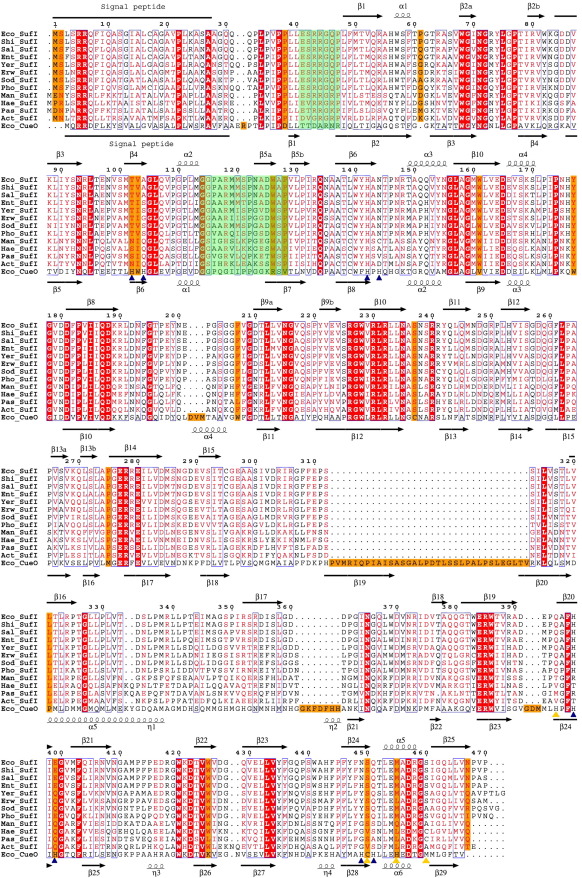
Sequence alignment of SufI proteins with CueO. Putative SufI sequences from various bacteria were identified with the computer program BLAST.^46^ The SufI sequences were aligned with one another and also with CueO from *E. coli* with ClustalW^47^ and coloured with WebESPript.^48^ Key: white character inside red box, strict identity; red character, similarity across SufI sequences; blue frame, similarity between conserved SufI sequences and CueO; orange box, differences between conserved SufI sequences and CueO. Two regions identified as having potential roles in SufI function are highlighted in green. Secondary structural elements for *E. coli* SufI and CueO are indicated above and below their respective sequences. Yellow triangles identify the type 1 copper centre coordinating residues in CueO, while blue triangles identify the residues in CueO that coordinate to the trinuclear copper centre. Eco, *Escherichia coli*; Shi, *Shigella flexneri*; Sal, *Salmonella typhimurium*; Ent, *Enterobacter* sp. 638; Yer, *Yersinia enterocolitica*; Erw, *Erwinia carotovora*; Sod, *Sodalis glossinidius*; Pho, *Photorhabdus luminescens*; Man, *Mannheimia succiniciproducens*; Hae, *Haemophilus influenzae*; Pas, *Pasteurella multocida*; Act, *Actinobacillus pleuropneumonia.*

**Fig. 7:**
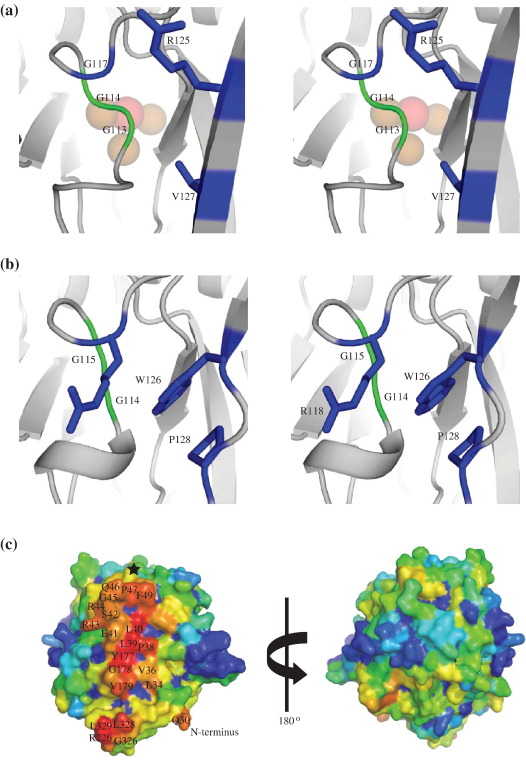
Identification and localization of conserved residues in SufI. (a) CueO: the conserved residues Gly113 and Gly114 (green sticks) sit above the trinuclear copper centre (shown as transparent spheres). Residues Gly117, Arg125 and Val127 (blue sticks) form the brim of a surface cavity that exposes the 112–114 loop (DGG in CueO, conserved as DGX across all multicopper oxidases) to the solvent. (b) SufI: in green, the glycine residues Gly114 and Gly115 corresponding to the glycines in (a); in blue, the residues Arg118, Trp126 and Pro128, which are all highly conserved across SufI sequences and prevent solvent access to the 112–114 loop in the SufI structure. (c) A surface representation of SufI, coloured red (surface residue most conserved) through orange, yellow, and green, to blue (least conserved). The N- and C-termini of the protein are shown for reference and highly conserved surface residues are labelled. A black star marks the location of the residues 118–128 that cover the pocket corresponding to the CueO catalytic centre. The right-hand panel shows a second view of the SufI surface, rotated with respect to the left-hand panel by 180° around the vertical axis. Images were generated using Pymol^40^ with (c) drawn using a PDB file produced by the WHISCY server.^49^

In an attempt to identify structural features that might be relevant to the specific function of SufI, we looked for residues that are conserved between SufI proteins from different organisms but are not found in CueO (Fig. 6). This approach identified two regions of particular note. The first, close to the N terminus at residues 41–47, is the motif ExRRGxP. Unfortunately, the density in this region, especially around the two arginines, is poor or missing in the models. This implies that the loop is very flexible, which could be important for any functional role it may play. The second region of interest is focussed on residues 118–128 in domain 1. Glycine residues at positions 114 and 115 of *E. coli* SufI are conserved in both SufI and CueO. In *E. coli* CueO, these glycine residues form part of a DGX motif conserved amongst all multicopper oxidases, which sits at the bottom of a surface-accessible region directly above the trinuclear copper centre (Fig. 7a) and is implicated in binding of dioxygen and, potentially, proton donation.^50,51^ However, in the SufI structure, the conserved residues arginine 118, tryptophan 126 and proline 128 are positioned in the space above the same glycines, occluding access from the surface of the molecule (Fig. 7b). This is additional evidence that the SufI molecule has lost the ability to accept ligands and/or substrates in the pocket that in CueO accommodates the Cu centre.

Using the WHISCY server,^49^ we examined the surface conservation of SufI residues in an attempt to identify regions of the molecular surface that may be involved in protein–protein interactions. We found a large number of conserved residues clustered to one face of SufI (Fig. 7c), including the conserved residues 41–47 discussed above (indicated by a black star in Fig. 7c). Given the role of SufI as a cell division protein and its localization to the septal ring during cell division, we speculate that this conserved face represents a binding site for interaction with other Fts proteins.

## Discussion

In this study, we show that both N- and C-terminal SufI/GFP fusions accumulate at the division site in constricting cells. Such localization is characteristic of Fts proteins and supports the proposal of Reddy and colleagues that SufI is a *bona fide* Fts protein.^7,13^ Interestingly, SufI localized to the septal ring regardless of whether NaCl was present in the growth medium, implying that SufI is not recruited to the ring in response to stress. The observation that *sufI* mutants are sensitive to stress probably reflects redundancy rather than a specific function for SufI in protecting the divisional apparatus from stress.^7^

Studies of Fts protein localization in various mutant backgrounds revealed that, at least in *E. coli*, the septal ring assembles via a largely linear pathway (reviewed in Refs. 3, 5 and 6). Here we have shown that SufI is a late recruit to the septal ring, as its localization depends on FtsZ, FtsQ, FtsL and FtsN. In this context, it is important to note that FtsN is the last known essential division protein recruited to the septal ring. Two additional observations also argue that SufI is a late recruit. First, septal localization was observed in only about half of the cells, which were growing with a doubling time of about 40 min. This percentage is similar to the ‘late’ proteins FtsN and AmiC.^34,52^ In contrast, ‘early’ proteins such as FtsZ and FtsA show septal localization in 70–90% of the cells.^53–55^ Second, almost all of the cells exhibiting localization were already constricting, which raises the intriguing possibility that an event associated with the onset of cytokinesis (rather than the mere presence of a protein in the septal ring) is involved in SufI recruitment.

SufI is the fourth soluble periplasmic protein shown to localize to the septal ring. The other three proteins are AmiC, EnvC and TolB.^34,36,56^ The latter is part of the trans-envelope Tol–Pal complex implicated in facilitating constriction of the outer membrane during cytokinesis.^56^ AmiC and EnvC are both peptidoglycan hydrolases.^36,57^ Localization of TolB and AmiC to septal ring assemblies requires FtsN, as reported here for SufI.^34,56^ Dependency studies have not been reported for EnvC, but given that it appears to join the septal ring prior to the onset of constriction, it is likely to localize ahead of SufI, AmiC and, perhaps, TolB.^56^ Indeed, it is tempting to speculate that peptidoglycan hydrolysis by EnvC contributes to forming the nascent constrictions that might be important for recruitment of SufI and AmiC to the division site.

While our results firmly establish that the physiological function of SufI is in cell division, the biochemical function remains to be determined. The structure of SufI, determined here at a resolution of 1.9 Å by X-ray crystallography, confirms that the protein belongs to the multicopper oxidase family. However, it also shows that SufI does not bind copper. Indeed, access to the region of the structure that forms the active site in multicopper oxidases is blocked in SufI by highly conserved residues. Together, these observations suggest that SufI is neither a copper-binding protein nor an enzyme. At present we favor the notion that SufI's role in cell division is to serve as a scaffolding protein that helps to maintain the coherence of the septal ring during constriction.

The ability of SufI overproduction to rescue a variety of *fts* mutants^7^ (S.J.R.A, E.P. and D.S.W., unpublished data) suggests that SufI engages in protein–protein interactions that contribute to the stability of the septal ring. Sequence analysis identified several conserved residues in SufI that were all found to map to the same face of the protein. This suggests that this face may form a binding surface for interactions with other Fts proteins at the septal ring. The osmotic remedial nature of the division defect in *sufI* null mutants could be explained if septal rings are somewhat unstable in the absence of SufI and if unfavorable ionic/osmotic conditions exacerbate that instability. With the insights gained from the structure in combination with the phenotypic assays described here and elsewhere,^7,13^ it should be possible to test these ideas by targeted mutagenesis of the conserved surface residues likely to be involved in protein–protein interactions.

SufI is used extensively as the model substrate in mechanistic studies of the Tat protein translocation pathway. Since the Tat apparatus translocates substrate proteins in the folded state, knowledge of the 3D structure of the substrate is a prerequisite for detailed studies of substrate-transporter interactions in this system. The structure of SufI reported here will allow rational site-specific labelling and engineering of SufI for use in the experimental analysis of Tat mechanism.

## Materials and Methods

### Strains and media

Strains used are listed in Table 4. CRIM vectors were integrated into the φ80 *att* site using the helper plasmid pAH123 as described.^61,62^ EC309 was constructed by P1 transduction with DRC14 as donor and EC251 as recipient. EC1908 was constructed by P1 transduction with TB54 as donor and EC251 as recipient. Strains were grown in LB with or without 10 g NaCl per liter (LB0N). Antibiotics were used at the following concentrations: ampicillin 200 μg/ml, chloramphenicol 30 μg/ml, kanamycin 40 μg/ml and spectinomycin 50 μg/ml for selection of plasmids and 35 μg/ml for chromosomal integrants.

### Plasmids

Plasmid pDSW932 (P_204_∷*sufI-gfp*, Amp^r^) was constructed by PCR amplification of *sufI* using chromosomal DNA as template and primers P985 (5′-caggaattctcactcagtcggcgtcag-3′) and P986 (5′-cctctgcaggttgttgttcggtaccggattgaccaacag-3′). The 1434-bp product was cut with EcoRI and PstI (sites underlined) and ligated into the same sites of pDSW208.^12^ Plasmid pDSW980 was created by digesting pDSW932 with SphI and ScaI to generate a 4454-bp fragment containing *lacI*^q^ P_204_∷*sufI-gfp*. This fragment was ligated to a 2668-bp fragment of the CRIM vector pJC69^61^ cut with SphI and HincII. A plasmid that expresses a *gfp-sufI* fusion was constructed in several steps. An 870-bp fragment carrying the Tat signal sequence coding region from *torA* and *gfp* was amplified by PCR using plasmid pTB6^36^ as template and primers P1074 (5′-cgggaattcaacaataacgatctctttcag-3′) and P1075 (5′-cgaagcttaggatccattgtatagttcatccatgcc-3′). This product was cut with EcoRI and HindIII (sites underlined) and ligated into the same sites of pDSW204^12^ to create pDSW962 (P_204_∷ss*torA-gfp*). A derivative of *sufI* lacking its Tat signal sequence was then amplified from plasmid pDSW932 with primers P1112 (5′-cggggatcccaacagcaaccgctacccgtt-3′) and P1113 (5′-ccgggatccttacggtaccggattgaccaa-3′). The 1344-bp product was cut with BamHI (sites underlined) and ligated into the same site of pDSW962. An isolate with the BamHI fragment in the desired orientation was designated pDSW978. The *lacI*^q^ P_204_∷*sstorA-gfp-sufI* was moved into a CRIM vector by ligating the 3170-bp SphI–ScaI fragment from pDSW962 into SpaI–HincII-digested pJC69 to create pDWS964. pDSW979 was created by ligating the 4502-bp SphI–ScaI fragment that carries *lacI*^q^ P_204_∷*sstorA-gfp-sufI* from pDSW978 into pJC69 cut with SphI and HincII. Plasmid pQE60-SufI directs the production of the *E. coli* SufI precursor protein with a C-terminal hexahistidine tag. The *sufI* gene was amplified from chromosomal DNA with primers SUFI1 (5′-gcgcgaattcgttttacatggagcaaatatg-3′) and SUFI2 (5′-gcgcagatctcggtaccggattgaccaacag-3′), digested with EcoRI and BglII (sites underlined) and cloned into the same sites in pQE60 (Qiagen) to give pQE60-SufI.

### Localization of fusions between GFP and SufI

Cultures were grown at 30 °C in LB with 1 mM IPTG to an OD_600_ of ∼ 0.5. Slides for visualizing live cells were prepared by pipetting 40 μl of molten 1% agarose (in water) onto a pre-warmed glass slide with two spacers made of laboratory tape. A second glass slide was placed on top to create a thin agarose pad. Once cooled, the two slides were separated and 5 μl of live culture in growth medium was applied to the agarose pad and covered with a #1 coverslip. Cells were immediately photographed at 100× magnification essentially as previously described.^63^ Typical exposure times for GFP were about 2 s. Digital images were imported to Adobe Photoshop for cropping and making minor adjustments to brightness and contrast. Final figures were assembled in Canvas.

For assessing the dependency of SufI-GFP on FtsZ, strains were grown in LB with 1 mM IPTG at 30 °C to mid log phase. Then 1 ml of culture was pelleted in a microcentrifuge and the cells were resuspended in 1 ml of LB0N. The washed cells were used at a dilution of 1:10 to inoculate 30 °C LB and 37 °C LB0N (both contained 1 mM IPTG). Cells were allowed to grow for 1.5 h, by which time the *ftsZ*(Ts) mutant in LB0N had become filamentous. Aliquots of the cultures were transferred to an agarose pad and photographed as described above.

For assessing dependency on FtsL, FtsQ and FtsN, the respective depletion strains carrying pDSW932 were grown in LB containing antibiotics, 0.2% l-arabinose and 10 μM IPTG. (The arabinose induces expression of the respective *fts* gene, while the IPTG induces expression of the *sufI-gfp* fusion.) Once the cultures reached mid log phase, 1 ml was pelleted in a microcentrifuge and the cells were resuspended in 1 ml of LB (for FtsL- and FtsQ-depletion strains) or 1 ml of LB0N (for the FtsN-depletion strain). The washed cells were used at a dilution of 1:50 to inoculate LB or LB0N containing antibiotics, 10 μm IPTG and either 0.2% arabinose or 0.2% glucose. Cultures were grown at 30 or 37 °C for 3–4 h, by which time the cells growing in the presence of glucose had become filamentous. Aliquots of the cultures were transferred to an agarose pad and photographed as described above.

To verify the periplasmic localization of SufI/GFP fusion proteins (Supplemental Fig. 1), transformants were grown at 30 °C in LB containing Amp and 10 μM IPTG to mid log phase. Aliquots of the cultures were transferred to an agarose pad and photographed as described above.

To examine localization of SufI and various division proteins in a *tat* null mutant (Supplemental Fig. 2), strain DADE was transformed with a series of *gfp* fusion plasmids. Transformants were grown at 30 °C in LB containing Amp to log phase. Then, aliquots of the cultures were transferred to an agarose pad and photographed as described above. IPTG was included at 10 μM only in the case of the *ftsZ-gfp* fusion; the remaining *gfp* constructs were adequately expressed in the absence of IPTG. Because some of the fusion constructs were not well tolerated in the DADE background, the length of the growth period was different for different strains. GFP, GFP-SufI, SufI-GFP and FtsZ-GFP overnight cultures were diluted 1:1000 and grown for 4 h. GFP-FtsI and GFP-FtsN overnight cultures were diluted 1:20 and grown for 1 h.

### Complementation of a Δ*sufI* mutant

Strains were grown at 37 °C in LB to mid log phase. Then an aliquot was harvested by centrifugation and the cell pellet was taken up in one-tenth volume LB0N with 1 mM IPTG. An aliquot of this suspension was used to inoculate LB0N with 1 mM IPTG and the culture was grown at 37 °C. Samples were removed periodically, fixed and photographed by phase contrast microscopy to analyze morphology. Cell lengths were measured with Image-Pro software version 4.1 (Media Cybernetics, Silver Spring, Md.).

### Immunoblotting

GFP-SufI and SufI-GFP were detected by immunoblotting as described.^64^

### Overproduction of SufI

Plasmid pQE60-SufI was used for the overproduction of C-terminally hexahistidine-tagged SufI. The plasmid was transformed into the Δ*tat* strain DADE harbouring pREP4; cells were grown at 37 °C with shaking in LB medium to mid log phase and induced by addition of IPTG to 2 mM final concentration. After 6 h further growth, cells were harvested by centrifugation (7000***g***, 15 min). Cell pellets were resuspended in 20 mM Mops (pH 7.2), 200 mM NaCl and 30 mM imidazole (buffer A) and disrupted by several passes through a French pressure cell at an operating pressure of 8000 psi. Cell debris was removed by centrifugation and the cleared lysate was applied to a nickel-charged, 5-ml HiTrap HP column (GE Healthcare) equilibrated in buffer A. SufI was eluted using a linear gradient of imidazole to 500 mM in buffer A. Fractions containing SufI, as assessed by SDS-PAGE, were pooled, concentrated using a 4-ml 30-kDa molecular mass cutoff Amicon Ultra (Millipore) and loaded onto a Superdex-75 10-300 column (GE Healthcare) equilibrated in 20 mM Mops (pH 7.2), 200 mM NaCl. The elution position of SufI from the column corresponded to an apparent molecular mass of approximately 55 kDa. N-terminal sequencing of the purified SufI showed that the first 17 residues corresponding to part of the signal peptide had been cleaved (data not shown).

### Crystallization and X-ray diffraction

Crystals were grown by the vapour-diffusion method in sitting drops at 20 °C. Screens were set up with a crystallization robot (TECAN, UK). Crystallization drops were obtained by mixing 0.2 μl of protein solution in 20 mM Mops (pH 7.2), 200 mM NaCl with 0.2 μl of the crystallization screen and were equilibrated against 100 μl of mother liquor. SufI crystals were grown by mixing the protein solution at 0.75 mg/ml with EB Wizard I condition 37 (2.5 M NaCl, 100 mM imidazole, pH 8.0). Crystals were visible after 5 days with dimensions of approximately 50 μm × 100 μm × 200 μm.

Diffraction data from the SufI crystals were collected at 100 K on beam lines ID23-1 and ID29 at the European Synchrotron Radiation Facility (ESRF), Grenoble, France. The crystals were cryoprotected in 8 M sodium formate. The crystals belonged to either space group *P*2_1_ or space group *P*2_1_2_1_2_1_, with both the monoclinic and orthorhombic crystals growing from the same condition, although the orthorhombic form required a longer growth period. All X-ray data integration and scaling were performed with the computer programs MOSFLM^65^ and Scala.^66^ The lattices of the two crystal forms were not found to be related. Table 3 gives the crystallographic data collection and processing statistics.

### Structure determination, model building and refinement

Initial structure factor phases were obtained for the orthorhombic SufI crystals by molecular replacement. The program SCWRL 3.0^67^ was used to model the SufI side chains onto the closely homologous blue copper oxidase CueO from *E. coli* (PDB ID 1PF3,^38^ 32% sequence identity) and the resulting model was used as a search model for molecular replacement with the computer program CCP4-PHASER.^66^ Two copies of the search model were found in the asymmetric unit and the SufI sequence was then built into the resulting density with X-fit,^68^ followed by multiple rounds of iterative refinement in BUSTER-TNT^69^ and model building in X-fit. Non-crystallographic symmetry (NCS) restraints were applied in BUSTER-TNT, with restraints lifted for individual residues where required. Once completed, the orthorhombic SufI structure was used as a search model for molecular replacement in the monoclinic crystal form, again with CCP4-PHASER.^66^ This provided a good solution with two copies in the asymmetric unit. The molecules were in a conformation very similar to those in the orthorhombic form with the exception of the loop around residues 55–66, which shifted between the two crystal forms. This region was removed from the search model and the molecular replacement search was repeated. Residues 55–66 were manually rebuilt into the resultant electron density with X-fit and then multiple rounds of iterative refinement with NCS in BUSTER-TNT, followed by model building in X-fit, were performed. The NCS for individual residues was lifted locally where required. Full details of the refinement parameters for both crystal forms can be found in Table 3. The anomalous difference density was calculated with the program CCP4-FFT^70^ and all superpositions were performed with the program CCP4-LSQKAB.^71^

### Protein Data Bank accession numbers

Coordinates and structure factors have been deposited in the PDB with accession numbers 2uxt and 2uxv.

## Figures and Tables

**Table 1:** *sufI* fusions to *gfp* rescue division

Genetic background	Fusion	Cell length, ave ± SD (μm)	% of cells > 10 μm
WT	*gfp*	4.7 ± 1.3	0.4
Δ*sufI*	*gfp*	8.8 ± 7.0	20.2
Δ*sufI*	*gfp-sufI*	6.1 ± 1.9	2.6
Δ*sufI*	*sufI-gfp*	4.5 ± 1.3	0.2

The strains used were EC1871, EC1874, EC1875 and EC1876. All were grown in LB0N at 37 °C, fixed and photographed under phase contrast microscopy. Length data are based on measurements of 500 cells for each strain.

**Table 2:** Localization of SufI

Genetic background	NaCl	Fusion	No. of cells scored	Cell length, ave ± SD (μm)	% of cells with rings
WT	+	*gfp-sufI*	122	5.5 ± 1.6	44
WT	+	*sufI-gfp*	184	3.9 ± 1.0	40
Δ*sufI*	+	*gfp-sufI*	302	5.6 ± 1.5	41
Δ*sufI*	+	*sufI-gfp*	216	5.0 ± 1.2	56
Δ*sufI*	−	*gfp-sufI*	201	6.2 ± 1.8	42
Δ*sufI*	−	*sufI-gfp*	208	4.8 ± 1.3	41

The strains used were EC1872, EC1873, EC1875 and EC1876.

**Table 3:** SufI X-ray data collection, processing and refinement

Data set	Orthorhombic	Monoclinic
A. *Data collection and processing*		
X-ray source	ESRF ID29-1	ESRF ID23-1
Detector	ADSC scanner	MAR scanner
Space group (*Z*)	*P*2_1_2_1_2_1_ (8)	*P*12_1_1 (4)
Unit cell parameters		
*a* (Å)	54.47	64.12
*b* (Å)	89.55	48.88
*c* (Å)	153.63	131.96
α (°)	90	90
β (°)	90	95.9
γ (°)	90	90
Wavelength	0.97370	0.87300
Resolution limits (Å)	77.4–1.9 (2.0–1.9)	64.0–2.6 (2.74–2.6)
Completeness (%)	98.4 (98)	99.5 (96.8)
Measured reflections	201,631	160,469
Unique reflections	58949	24830
Multiplicity	3.4 (2.9)	6.5 (5.7)
*R*_merge_	0.083 (0.397)	0.154 (0.425)
*I*/σ(*I*)	5.2 (1.9)	3.4 (1.6)

B. *Refinement*		
Crystal	Orthorhombic, *P*2_1_2_1_2_1_	Monoclinic, *P*12_1_1
Resolution range	39.8–1.9 (2.0–1.9)	59.8–2.6 (2.74–2.6)
*R*_all_ (%)	19.3 (20.9)	21.6 (26.2)
*R*_work_ (%)	19.1 (20.8)	21.4 (26.1)
*R*_free_ (%)	22.9 (24.1)	24.6 (28.0)
rmsd bond lengths (Å)	0.005	0.005
rmsd bond angles (°)	0.94	1.2
Residues modelled (range)	Chain A: 422 (30–295, 312–330, 335–471)	Chain A: 422 (30–295, 314–469)
	Chain B: 420 (31–43, 46–295, 313–470)	Chain B: 422 (29–295, 315–469)
Water molecules modelled	Chain A: 219	Chain A: 34
	Chain B: 193	Chain B: 28
Average B (protein) (Å^2^)	Chain A: 28.9	Chain A: 32.7
	Chain B: 31.5	Chain B: 32.3
Average B (water molecules) (Å^2^)	Chain A: 35.9	Chain A: 22.3
	Chain B: 37.6	Chain B: 24.0
Residues in favoured regions of Ramachandran plot (%)	95.3	88.9
Residues in forbidden regions of Ramachandran plot (%)	0.2	1.9
PDB identifier	2UXT	2UXV

Values for the highest-resolution shell are given in parentheses.

**Table 4:** Strains and plasmids

Strain or plasmid	Relevant features	Source or reference
*Strain*		
DADE	MC4100 Δ*tatABCD*Δ*tatE*	Wexler *et al.*^58^
DRC14	MC4100 *ftsZ84*(Ts) *leu*∷Tn*10*	D. RayChaudhuri
EC251	K12 wild-type MG1655	Arends and Weiss^59^
EC309	EC251 *ftsZ84*(Ts) *leu*∷Tn*10*	This study
EC839	K12 wild-type BW25113	Baba *et al.*^35^
EC1751	BW25113 *sufI<>aph* Kan^r^	Baba *et al.*^35^
EC1871	EC839 *attP*φ 80∷pDSW964(P_204_∷ss*torA*-*gfp*)	This study
EC1872	EC839 *attP*φ 80∷pDSW979(P_204_∷ss*torA*-*gfp*-*sufI*30–470)	This study
EC1873	EC839 *attP*φ 80∷pDSW980(P_208_∷*sufI*-*gfp*)	This study
EC1874	EC1751 *attP*φ 80∷pDSW964(P_204_∷ss*torA*-*gfp*)	This study
EC1875	EC1751 *attP*φ 80∷pDSW979(P_204_∷ss*torA*-*gfp*-*sufI*30–470)	This study
EC1876	EC1751 *attP*φ 80∷pDSW980(P_204_∷*sufI*-*gfp*)	This study
EC1908	EC251 pBAD∷*ftsN* (Kan^r^)	This study
EC2065	EC309 *attP*φ 80∷pDSW980(P_204_∷*sufI*-*gfp*)	This study
JMG265	KS272 *ftsL*∷Tn*phoAL81* IS *50R* (Kan^r^)/pBAD33-LLL	J. M. Ghigo
JOE170	KS272 *ftsQ*∷Tn*phoA50* (Kan^r^)/pJC10	Chen *et al.*^60^
TB54	DY329 P_ftsN_ <> (aph araC P_BAD_)	Bernhardt and de Boer, 2001^36^
XL1-Blue	*recA1 endA1 gyrA96 thi-1 hsdR17 supE44 relA1 lac* [F′ *proAB* l*acI^q^ Z*Δ*M15* Tn*10* (Tet^r^)]	Stratagene

*Plasmid*		
pTB6	*lacI*^q^ P_lac_∷ss*torA-gfp-*T7 tag, Amp^r^	Bernhardt and de Boer^36^
pJC69	oriR_R6Kγ_, *attP*φ80, Spc^r^, CRIM based plasmid	Chen and Beckwith^61^
pAH123	λcI*857*, *rep101*ts origin, *Pr-int*φ80, CRIM helper plasmid Amp^r^	Haldimann and Wanner^62^
pDSW204	pTrc99A based, promoter down mutation in − 35 region (P_204_ promoter), Amp^r^, ColE1 *ori*	Weiss *et al.*^12^
pDSW207	*gfp* fusion vector derived from pDSW204, Amp^r^	Weiss *et al.*^12^
pDSW208	*gfp*-fusion vector derived from pDSW204, Amp^r^	Weiss *et al.*^12^
pDSW210	*gfp*-fusion vector with promoter down mutations in both the − 35 and − 10 regions (P_206_ promoter), ColE1 *ori*, Amp^r^	Weiss *et al.*^12^
pDSW231	pDSW210-*ftsZ* (P_206_∷*ftsZ-gfp*, Amp^r^)	Laboratory collection
pDSW234	pDSW207-*ftsI* (P_204_∷*gfp-ftsI*, Amp^r^)	Weiss *et al.*^12^
pDSW238	pDSW207-*ftsN* (P_204_∷*gfp-ftsN*, Amp^r^)	Laboratory collection
pDSW932	pDSW208-*sufI* (P_204_∷*sufI-gfp*, Amp^r^)	This study
pDSW962	pDSW204-ss*torA-gfp* (P_204_∷ ss*torA-gfp*, Amp^r^)	This study
pDSW964	pJC69-derivative, *lacI*^q^, P_204_∷ss*torA*-*gfp*, Spc^r^	This study
pDSW978	pDSW962-*sufI* (P_204_∷ ss*torA-gfp-sufI*30–470, Amp^r^)	This study
pDSW979	pJC69-derivative, *lacI*^q^, P_204_∷ss*torA*-*gfp*-*sufI*30–470, Spc^r^	This study
pDSW980	pJC69-derivative, *lacI*^q^, P_204_∷*sufI*-*gfp*, Spc^r^	This study
pQE60-SufI	pQE60 *sufI*_*His*_, Amp^r^	This study

## Appendix A

Supplementary Table 1 Localization of SufI-GFP in *fts* mutants.
