# Supplementary material for: The Escherichia coli Cell Division Protein and Model Tat Substrate SufI (FtsP) Localizes to the Septal Ring and Has a Multicopper Oxidase-Like Structure
Source: J Mol Biol. 2009 Feb 20;386(2):504–19. doi: 10.1016/j.jmb.2008.12.043 (PMC2661564; doi:10.1016/j.jmb.2008.12.043)
Supplement: Supplementary — Table 1 Localization of SufI-GFP in fts mutants. [file mmc1.pdf]

Supplementary Table 1. Localization of SufI-GFP in *fts* mutants

Genetic Background[a]	Growth Conditions			No. of cells scored	Cell Length, Ave ± SD (μm)	No. of SufI-GFP rings	% of cells with rings	Ring spacing[b]
	Temp	NaCl	Sugar					
WT	30	+	-	195	4.2 ± 1.0	92	47	9.0
	37	-	-	311	5.0 ± 1.2	140	45	11
FtsZ(TS)	30	+	-	272	6.2 ± 1.7	109	36	16
	37	-	-	186	24.2 ± 8.3	1	1	4500
FtsQ(dep)	30	+	Ara	235	6.7 ± 3.0	116	49	14
	30	+	Glu	146	34.3 ± 11.5	4	3	1300
FtsL(dep)	30	+	Ara	243	6.8 ± 2.0	106	44	16
	30	+	Glu	216	22.1 ± 6.6	4	2	1200
FtsN(dep)	37	-	Ara	139	5.5 ± 1.4	63	45	12
	37	-	Glu	152	25.1 ± 10.8	24	16	160

[a] The strains used were EC1873, EC2065, JOE170/pDSW932, JMG265/pDSW932, and EC1908/pDSW932.

[b] Sum of cell lengths divided by the total number of rings, rounded to two significant figures.
