# Supplementary figures and images for: The Escherichia coli Cell Division Protein and Model Tat Substrate SufI (FtsP) Localizes to the Septal Ring and Has a Multicopper Oxidase-Like Structure
Source: J Mol Biol. 2009 Feb 20;386(2):504–19. doi: 10.1016/j.jmb.2008.12.043 (PMC2661564; doi:10.1016/j.jmb.2008.12.043)

torA<sup>SS</sup>-GFP

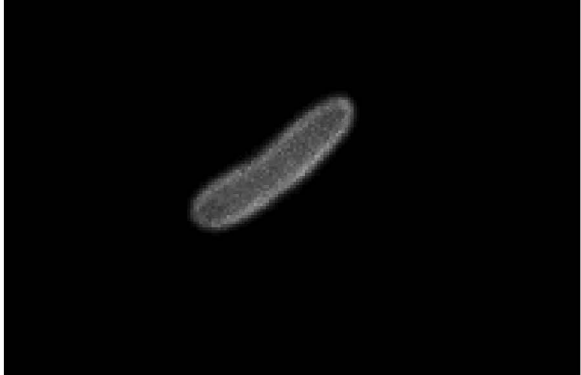

SufI-GFP

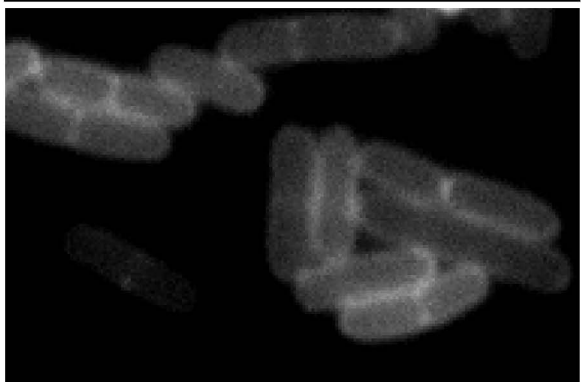

torA<sup>SS</sup>-GFP-SufI

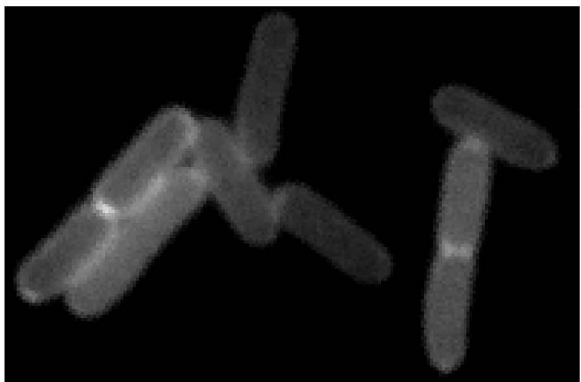

Supplement: Supplementary Figure [file mmc2.pdf]

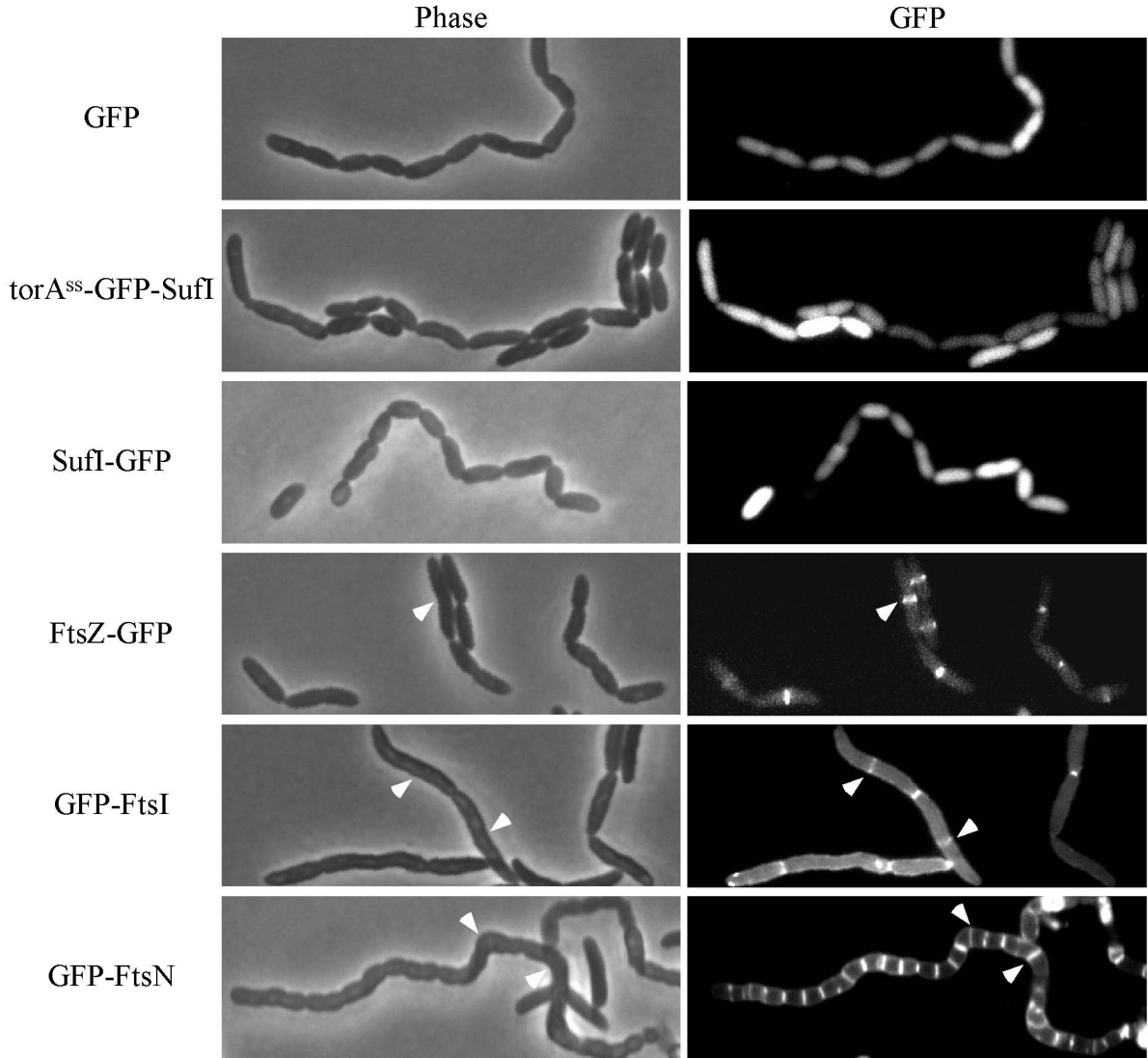

Supplement: Supplementary Figure [file mmc3.pdf]
